# Multiple extradural spinal arachnoid cysts: a case report and review of the literature

**DOI:** 10.1186/1757-1626-2-7531

**Published:** 2009-05-20

**Authors:** Mohammad Ali Bitaraf, Mehdi Zeinalizadeh, Ali Tayebi Meybodi, Keyvan Tayebi Meybodi, Zohreh Habibi

**Affiliations:** Department of Neurosurgery, Imam Khomeini Hospital, Tehran University of Medical SciencesTehran 14197Iran

## Abstract

Extradural spinal arachnoid cysts are rare lesions, which may become symptomatic due to mass effect. Multiple cysts are even rarer of which few are reported to date. A 17-year-old male with acute onset urinary retention and progressive paraparesis is presented. Magnetic resonance imaging of spine revealed multiple spinal extradural arachnoid cysts located dorsal to the spinal cord, causing mass effect. The patient underwent surgery for excision of the cyst and closure of dural defects. He gained urinary continence and near normal muscle strength of lower extremities over a period of two weeks following operation. Up to date, there have been only sixteen reported cases of multiple spinal extradural arachnoid cysts in the literature and the present case appears to be the second most extensive one reported so far. Appreciation of the rarity of such lesions as well as the importance of surgical planning (especially pre-operative localization of the dural defects) is highlighted.

## Introduction

The term “spinal arachnoid cyst” is a relatively rare entity among spinal lesions. According to some authors, extra-dural cysts are less common than intra-dural ones [1], but this is not believed by many others [2,3]. Symptomatic extra-dural arachnoid cysts are even less common [4]. To the best of our knowledge, only sixteen cases of multiple extra-dural arachnoid cysts have been reported in the literature [1,4-9]. In this report, the authors present a case of multiple extra-dural arachnoid cysts (five separate cysts from T4 to L5) in a 17-year-old male presenting with spastic paraparesis. Surgical intervention and post-operative course are presented and discussed.

## Case presentation

**Figure 1.**
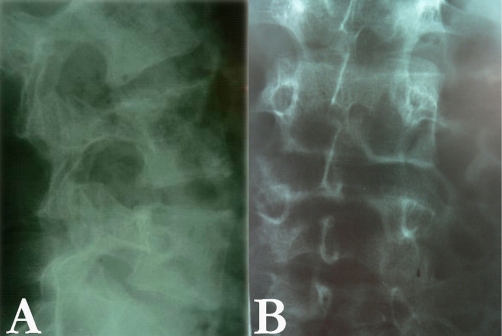
Lateral (**A**) and antero-posterior (**B**) X-ray studies of the spine showing increased interpeduncular space with vertebral body scalloping all through the upper thoracic to lower lumbar region. Note the preoperative kyphosis of the thoracic spine in the right panel, along with thin bony trabeculae confirming the fragile laminae.

**Figure 2.**
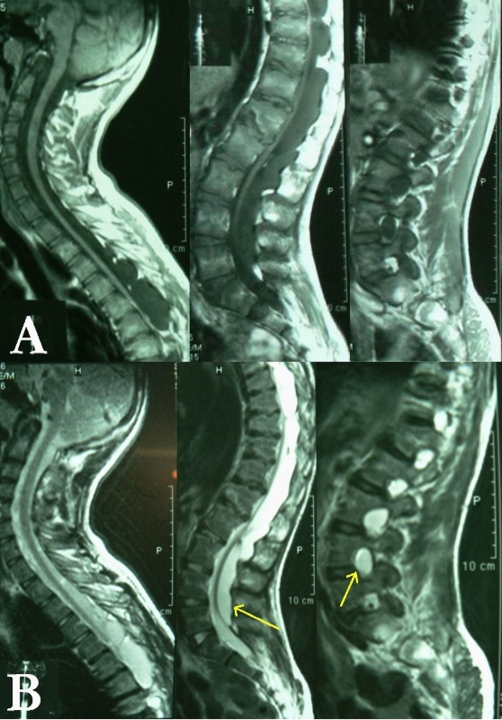
T1 (**A**) and T2-weighted (**B**) sagittal MRI of the cervical and thoraco-lumbar spine with gadolinium-DTPA injection disclosing non-enhancing multiple extra-dural cystic lesions with severe compressive effect upon the thinned ventrally compressed cord. Arrows point to the most caudal cyst extending down to L5 with enlarged intervertebral foramina.

**Figure 3.**
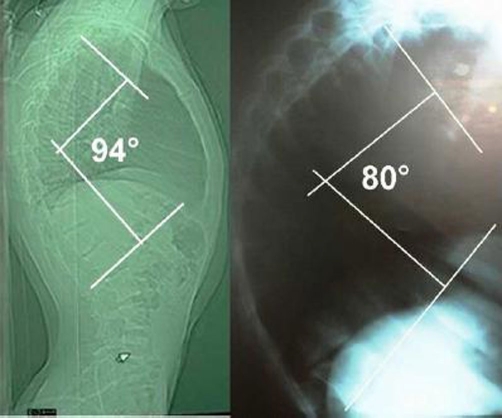
Pre- and post-operative lateral X-rays of spine with the measurement of kyphosis angle showing increased kyphotic deformity.

A 17-year-old Caucasian male of Iranian nationality was visited at our institution complaining of forced urination and hesitancy since last two weeks which evolved to frank urinary retention on admission day 2. One month before presentation, he developed progressive weakness of both lower limbs. Neurological examination revealed the following: spastic paraparesis (lower limb forces: 1/5 proximally and 0/5 distally); sensory level with hypesthesia to pinprick, light touch, and loss of vibration sense up to T2 level. Figures 1, 2 and 3 depict plain X-ray and MRI films of the spine.

A T4 to L5 laminectomy (sparing the T5 lamina) was performed disclosing multiple extra-dural cystic lesions. They were filled with CSF-like fluid. The cysts communicated with the subarachnoid spaces along the dural sleeves of the following nerve roots: (1) right T4, (2) left T6, (3) left T10, (4) left T11, (5) left L2. The cysts were excised and their ostia were closed. Closure was enforced in some points with muscle flaps.

Microscopic examination of the specimens revealed fibrocollagenous tissue with an inner layer of arachnoid. The early postoperative course passed uneventfully. The patient regained urinary continence and was ambulatory with 4+/5 lower extremity forces over a period of two weeks following operation. The patient was symptom free during 12-month period of follow-up. Yet, kyphosis, a complication of the procedure, evolved and progressed during the follow up period which was controlled by brace and its progression ceased.

## Discussion

Spinal arachnoid cysts were first described by Spiller in 1903 [8], although the first reported case is traced back to 1898 by Nonne (an autopsy finding) [5]. Most of the reported cases are solitary ones [5]. Extra-dural cysts occur most frequently in the thoracic spine (65%) followed by lumbar and lumbo-sacral (13%), thoraco-lumbar (12%), sacral (6.6%), and cervical (3.3%) regions. They could occur either dorsal or ventral to the cord, with the former being more common.

Regarding the reported case so far, multiple extra-dural spinal arachnoid cysts seem to occur with higher frequency in females (Table 1). The peak age of presentation is the early second decade of life. Thoracic cysts usually occur in young adolescents whereas thoraco-lumbar and lumbar cysts usually appear in adults in the 3-4^th^ decade of life. This may be due to relatively smaller diameter of spinal canal in thoracic region. Lower thoracic, higher thoracic, and thoraco-lumbar regions are involved in order of reducing frequency [1,4-8]. Features of the reported cases are summarized in Table 1.

**Table 1.** Summary of reported cases of multiple extradural spinal arachnoid cysts.

Age/Gender	Number/Level of cysts	Symptoms/Duration	Treatment/Outcome	Complications	Author/Reference number
42/F	3/L1-3	NA/15 yrs	Laminectomy; cystectomy/NA	NA	Dutoit*/5
15/M	2/T6-11	NA/NA	Laminectomy; cystectomy/NA	NA	Lombardi and Passerini*/5
29/F	3/L1-4	NA/4 yrs	Laminectomy; cystectomy/NA	NA	Nugent*/5
13/F	2/T6-12	NA/14 mo	Laminectomy; cystectomy/NA	NA	Strang and Tovi*/5
15/M	2/T7-11	NA/6 wks	Laminectomy; cystectomy/NA	NA	Dastur*/5
13/F	4/T4-12	NA/2 mo	Laminectomy; cystectomy/NA	NA	Kronborg*/5
11/F	3(intradural), 3(extradural)T1-L2	NA/NA	Laminoplasty; as much as possible excision (intradural); cystectomy (extradural)/NA	KS	Otake S*/8
9/F	6/T2-S1	Progressive gait disturbance/2 wks	T1-L2 laminoplasty; cystectomy; cyst within lumbar and sacral canal was left untreated/incomplete recovery	K	Myles*/8
12/M	11/T2-T12	NA	Laminoplasty; cystectomy and closed communication; cystectomy and unclosed communication; cyst-abdominal shunt/recurrence of the cyst in which the communication was not closed	K	Obara K*/8
11/M	5/T5-L5	Gait disturbance/1 mo	Laminectomy; cystectomy and closed communication; cyst at the cauda equinal level was left untreated/complete recovery	KS	Takagaki/8
12/F	2/L1-2	Progressive intermittent bilateral claudication/6 mo	Laminectomy; cystectomy; dural repair/complete recovery	-	Chang/4
12/M	2/T5-10	Progressive paraparesis; unsteady gait; urinary hesitancy/10 mo	Laminotomy, cystectomy; neck sutured/complete recovery	-	Kanaan/6
14/M	2/T4-11	Progressive paraparesis/4 mo	Laminotomy, cyst excision, repair of dural defect/complete recovery	-	Suryaningtyas/1
31/F	NA/T7-L3	Acute paraplegia/NA	Excision of the posterior wall of the symptomatic lesion/NA	NA	Marbacher/7
12/F	4/T5-12	Frequent stumbling/2 yrs	Laminectomy and laminoplasty; cystectomy; stalk ligation; dural defect repair	-	Yabuki/9
13/F	3/T5-L5	Back pain/4 mo	Laminoplasty; cystectomy; stalk ligation	-	Yabuki/9
17/M	5/T4-L5	Progressive paraparesis; urinary hesitancy/1 mo	Laminectomy; cystectomy; stalk ligation; enforcement with muscle flap/complete recovery	K	Current case
					

K, kyphosis; S scoliosis; KS kyphoscoliosis; NA, Not Available. Asterisks refer to the cases not originally available to the authors.

With the advent of new diagnostic modalities and using state-of-the-art technologies, diagnosis of these complex lesions is not a dilemma at present. Formerly, plain X-ray studies and myelography led to clues for detection of the cysts. Plain radiographs have not been useful in the diagnosis of spinal cysts except for implicit signs such as enlarged spinal canal, bony erosions of the spine including eroded pedicles and posterior elements, slender pedicles, widened foramina, increased inter-peduncular space, and posterior vertebral body and laminar scalloping, and kypho-scoliosis (Figure 1) [3-4,10-11].

MRI is the technique of choice for both diagnosis and delineation of the cyst dimensions. Identification of a sharp interface between the cyst and subarachnoid space related to the cyst wall and the surrounding dura mater, particularly on T2-weighted MR images, allows easy diagnosis of an extra-dural arachnoid cyst [2]. With all sequences, the signal within the lesion is iso-intense to CSF [12]. MRI can also show the extent of cord atrophy and myelo-malacia. Epidural fat capping of the lesion at its superior and inferior poles can be seen on sagittal T1-weighted MR images, which further suggests its extra-dural location [11].

One important aspect of preoperative evaluation is to determine the size and location of the dural defect, since it facilitates its repair via minimal laminectomy [13]. However, the preoperative identification of dural defects is difficult [14]. Myelography and CT myelography may disclose the cystic nature of the lesion along with its communication with the subarachnoid space as well as determination of the communication between different cysts [10]. Myelography may show a filling defect at the dural diverticula [11]. But, according to Liu et al [11], filling of the cyst through the communicating pedicle may not always be visualized by myelography with or without CT myelography, particularly if the patient is placed prone. Some authors have used cinematic MRI to show the dural defect [14,15]. Miyamoto et al have shown MR-myelogram to be a useful technique in identification of the dural defect [13].

Total cyst excision, obliteration of the communicating pedicle, and repair of the dural flaw is the treatment of choice of the symptomatic lesions. Postoperative kyphosis may be prevented by performing laminoplasty rather than laminectomy [6]. Nevertheless, there is paucity of evidence proving the superiority of laminoplasty to laminectomy [16]. It needs not to be over-emphasized that when possible, the surgical approach should proceed so as to address the dural defects directly in order to avoid extensive laminoplasty or laminectomy, to prevent post-operative spinal deformity. According to the reported cases in the literature, this is obviously not possible in all circumstances. As it is seen in the pre-operative imaging studies of our patient, the laminae are very thin and fragile and therefore not amenable to a laminoplasty procedure. A search of the literature also did not disclose any particular and proven superiority of laminoplasty or laminectomy plus posterior fusion against laminectomy [16]. Regarding the patient's age (late puberty) it seemed rational to follow the patient both clinically and radiologically and perform a fixation if a debilitating deformity ensued (fortunately no major disability occurred despite kyphotic deformity which was controlled by brace, although the kyphosis progressed for a period of time). Furthermore, to the authors' knowledge, all reported multiple extra-dural arachnoid cysts treated with laminoplasty (except one 13-year-old female) were complicated by kyphosis and/or scoliosis, therefore a firm relationship between laminoplasty and reduced rate of kyphosis deformity could not be deduced by the current evidence.

## Conclusion

Multiple extra-dural arachnoid cysts are rare lesions. To the best of our knowledge, only sixteen extra-dural variants of these lesions are reported previously in the literature. The present case is the second most extensive case of multiple cysts reported to date. Reasonable addressing such pathology requires correct and comprehensive preoperative evaluation, along with a logical operative approach chosen. Since laminectomy and laminoplasty (in multiple levels) might lead to post-operative spinal deformity, it seems that the best approach would be exact determination of the communication site between the cyst and the subarachnoid space, which is currently suggested to be done through Myelography with and without CT [10]. Although spinal instrumentation seems to be a reasonable approach after spinal deformity ensues, if correct determination of communication site(s) is done pre-operatively, this would not be a problem. Patient follow up is especially important regarding possible recurrence and complications.

## List of abbreviations

CSF: Cerebrospinal fluid
CT: Computed tomography
MRI: Magnetic resonance imaging
